# The p52 isoform of SHC1 is a key driver of breast cancer initiation

**DOI:** 10.1186/s13058-019-1155-7

**Published:** 2019-06-15

**Authors:** Kevin D. Wright, Bradley S. Miller, Sarah El-Meanawy, Shirng-Wern Tsaih, Anjishnu Banerjee, Aron M. Geurts, Yuri Sheinin, Yunguang Sun, Balaraman Kalyanaraman, Hallgeir Rui, Michael J. Flister, Andrey Sorokin

**Affiliations:** 1 Cardiovascular Center, Medical College of Wisconsin, 8701 Watertown Plank Road, Milwaukee, WI 53226 USA; 2 Department of Medicine, Medical College of Wisconsin, 8701 Watertown Plank Road, Milwaukee, WI 53226 USA; 3 Department of Physiology, Medical College of Wisconsin, 8701 Watertown Plank Road, Milwaukee, WI 53226 USA; 4 Institute for Health and Equity, Medical College of Wisconsin, 8701 Watertown Plank Road, Milwaukee, WI 53226 USA; 5 Department of Pathology, Medical College of Wisconsin, 8701 Watertown Plank Road, Milwaukee, WI 53226 USA; 6 Department of Biophysics, Medical College of Wisconsin, 8701 Watertown Plank Road, Milwaukee, WI 53226 USA; 7 Free Radical Research Center, Medical College of Wisconsin, Milwaukee, WI 53226 USA

**Keywords:** DMBA, Shc proteins, Breast cancer, Signaling, Rat model

## Abstract

**Background:**

SHC1 proteins (also called SHCA) exist in three functionally distinct isoforms (p46SHC, p52SHC, and p66SHC) that serve as intracellular adaptors for several key signaling pathways in breast cancer. Despite the broad evidence implicating *SHC1* gene products as a central mediator of breast cancer, testing the isoform-specific roles of SHC1 proteins have been inaccessible due to the lack of isoform-specific inhibitors or gene knockout models.

**Methods:**

Here, we addressed this issue by generating the first isoform-specific gene knockout models for p52SHC and p66SHC, using germline gene editing in the salt-sensitive rat strain. Compared with the wild-type (WT) rats, we found that genetic ablation of the p52SHC isoform significantly attenuated mammary tumor formation, whereas the p66SHC knockout had no effect. Rats were dosed with 7,12-dimethylbenz(a)anthracene (DMBA) by oral gavage to induce mammary tumors, and progression of tumor development was followed for 15 weeks. At 15 weeks, tumors were excised and analyzed by RNA-seq to determine differences between tumors lacking p66SHC or p52SHC.

**Results:**

Compared with the wild-type (WT) rats, we found that genetic ablation of the p52SHC isoform significantly attenuated mammary tumor formation, whereas the p66SHC knockout had no effect. These data, combined with p52SHC being the predominant isoform that is upregulated in human and rat tumors, provide the first evidence that p52SHC is the oncogenic isoform of *Shc1* gene products in breast cancer. Compared with WT tumors, 893 differentially expressed (DE; FDR < 0.05) genes were detected in p52SHC KO tumors compared with only 18 DE genes in the p66SHC KO tumors, further highlighting that p52SHC is the relevant SHC1 isoform in breast cancer. Finally, gene network analysis revealed that p52SHC KO disrupted multiple key pathways that have been previously implicated in breast cancer initiation and progression, including ESR1 and mTORC2/RICTOR.

**Conclusion:**

Collectively, these data demonstrate the p52SHC isoform is the key driver of DMBA-induced breast cancer while the expression of p66SHC and p46SHC are not enough to compensate.

**Electronic supplementary material:**

The online version of this article (doi:10.1186/s13058-019-1155-7) contains supplementary material, which is available to authorized users.

## Background

The progression of breast carcinomas is controlled by the aberrant expression of a variety of signaling molecules expressed in the mammary tumor itself, or in cells of the tumor microenvironment [1, 2]. The principal factors that control mammary tumor formation remain unclear and insufficiently defined. Mediating some of these signals are the SHC adaptor proteins. Many studies have implicated SHC1 involvement in signaling by epidermal growth factor receptor-2 (HER-2), estrogen receptor (ER), and prolactin (PR) signaling, three well-established biological markers for prognosis and response to breast cancer therapy [3–5]. More recently, increased expression of the p66SHC isoform was shown to occur in cell lines with increased metastatic potential and in patient samples with metastatic breast tumors [5–7]. However, to date, there has been no direct in vivo examination of the role of specific SHC1 protein isoforms in the progression of mammary tumorigenesis.

The ubiquitously expressed adaptor protein SHC1 [8] exists in three isoforms with relative molecular masses of 46 kDa, 52 kDa, and 66 kDa (p46SHC, p52SHC, and p66SHC) encoded by single gene *SHC1* (also known as *SHCA*). p46SHC and p52SHC arise from the use of alternative translation initiation sites within the same transcript, whereas p66SHC isoform contains a unique N-terminal region and is believed to be generated as a result of activation of an alternative promoter [9]. Although all three Shc proteins contain a recognition site for the SH2 domain of the adaptor protein GRB2, a number of studies have suggested distinct physiological roles for the three isoforms [10–12]. Additionally, different intracellular localization patterns have been reported for each (reviewed in [12]). Moreover, it has been shown that p66Shc and p46Shc/p52Shc exert counter-regulatory effects on the *c-fos* promoter [11]. This has given rise to the general consensus that isoform p52SHC functions as an adaptor protein linking receptor tyrosine kinases and G-protein-coupled receptors with the RAS signaling cascade, while p66SHC isoform contributes to reactive oxygen species (ROS) production as well as regulation of vascular contraction [12]. While early studies suggested p46SHC functions to be like p52SHC, recent data indicate a distinct role for p46SHC in the mitochondria [12, 13].

In mice, the polyomavirus middle T antigen (MT) model is frequently used to study mammary tumor progression. Several lines of evidence suggest the direct involvement of products of the *Shc1* gene in the regulation of breast cancer initiation and progression in this model [14, 15]. *Shc1* products have been shown to directly bind to the MT antigen protein, serving as an adaptor protein leading to activation of downstream signaling cascades such as RAS/MAPK and PI3K, all of which have a positive effect on tumorigenesis [14]. Furthermore, increased expression of *Shc1* gene products have been shown to accelerate tumorigenesis in the MT antigen model, indicating its importance in this model of breast cancer [16]. More recently, studies have shown that *Shc1* gene products in this model further function in a paracrine manner to promote a pro-tumorigenic state by inhibiting the adaptive immune response [17, 18]. These data clearly indicate both cell autonomous function, as well as microenvironment effects of *Shc1* products in this model of mammary tumorigenesis. However, in these studies, the role of individual SHC1 isoforms have not been tested, as many of the mutations in the previous studies will alter all three Shc1 protein isoforms, or overexpression systems will produce multiple SHC1 protein isoforms. Furthermore, this is an activated model in which increased expression of several well-known signaling pathways promotes tumorigenesis. This model does not address the role of SHC1 proteins in spontaneous lesions and tumorigenesis that is more typical of human populations that develop mammary cancer.

For these reasons, we generated the first animal models for *Shc1*-isoform-specific knockout of the p52SHC (p52SHCKO) isoform and first rat knockout of p66SHC (p66SHCKO) isoform [19] in the SS/JrHsdMcwi (SS) genetic background, which is a well-studied rat parental background for breast cancer risk [20, 21]. Mammary tumors were induced by the oral administration of 7,12-dimethylbenz(a)anthracene (DMBA), a known inducer of breast cancers that introduce random mutations into the genome and develop spontaneous mammary tumors [22–24]. DMBA-induced tumors are typically ER+ and PR+ lesions and serve as excellent models to study these types of tumors, as these represent a clinically needed model [25]. In humans, ER+ tumors represent tumor types successfully targeted by hormone therapy; however, few clinical options remain in case of cancer relapse. Accordingly, analysis of tumor incidence, latency, and molecular characteristics of mammary tumors in the rat DMBA model could provide data relevant for breast cancer treatment in humans. Here, we show for the first time that p52SHC is the predominant *Shc1* isoform that mediates mammary tumorigenesis via multiple key signaling pathways, whereas the p66SHC isoform is expendable in DMBA-induced tumorigenesis.

## Methods

### Animals

All studies were performed on rats generated and maintained on the SS/JrHsdMcwi (SS) genetic background. Generation of the p66SHC-KO rats has been described previously [19]. Briefly, p52SHC knockout rats (SS-*Shc1*^*em6Mcwi*^) were generated by pronuclear injection of a CRISPR plasmid expressing Cas9 and single-guide RNA targeting the sequence CACTCAGCTTGTTCATGTCCTGG (protospacer adjacent motif underlined) into one-cell SS (SS/JrHsdMcwi) rat embryos. Founder animals were genotyped by Cel-1 assay and confirmed by Sanger sequencing to identify a founder animal harboring a 6-bp deletion including the p52SHC initiation codon. All subsequent litters are genotyped using the following primers: p52ShcKO forward 5′-CCCTCCTCCAGGACAAGC-3′; wild-type forward 5′-TCCTCCAGGACATGAACAAGC-3′; common reverse 5′-TATGCACTCACCCGAACCAA-3′. p52SHC-KO rats are born in the expected genotypic ratio with normal litter size and able to breed as homozygous knockouts. All rats were fed ad libitum on a low salt (0.4% NaCl, Teklad7034) diet. Animal use and welfare procedures adhered to the NIH Guide for the Care and Use of Laboratory Animals following protocols reviewed and approved by the Medical College of Wisconsin Institutional Animal Care and Use Committee.

### Mammary tumor model

To induce mammary tumors, 45- to 55-day-old female rats (WT *n* = 14, p52SHC-KO *n* = 12, p66SHC-KO *n* = 13) were administered 65 mg/kg of body weight of 7,12-dimethylbenz(a)anthracene (DMBA) by oral gavage, as described [20]. Animals were then palpated weekly for tumor formation in the mammary fat pads. The latency of tumor development is defined as the time between DMBA administration and detection of the first tumor, whereas tumor multiplicity is the number of tumors per rat at week 15. At week 15 post-DMBA, rats were euthanized by CO_2_ asphyxiation and number of tumors and total tumor burden per rat were recorded. Tumor burden is calculated as the sum total weight of all tumors from one animal.

### Whole-mount stain of mammary glands

Carmine alum staining of whole inguinal mammary glands was performed using standard protocols. Briefly, excised fourth and fifth mammary glands from the left and right side of adult approximately three-month-old rats were stretched and pressed onto a 2 × 3 in. glass slide and fixed 24 h in Carnoy’s fixative. Following rehydration to H_2_O, slides were stained in Carmine Alum stain 24 h and then serially dehydrated in ethanol. Finally, slides were cleared in Histo-Clear™ clearing reagent up to 72 h until translucent and coverslipped using Histomount mounting media. Images were taken on a Nikon SMZ1500 microscope.

### RT-qPCR

Quantitative real-time PCR was performed using Power SYBR™ Green PCR Master Mix (Invitrogen) on a TissueScan cancer survey cDNA panel (OriGene), covering 8 different cancers, with three normal tissue controls and 9 samples for each type of cancer included. Primers were validated for efficiency using cDNA from human mesangial cell cultures, known to express all three SHC protein isoforms. Additionally, dissociation curve analysis and gel electrophoresis of PCR products were used to validate specificity. Primer sequences were p52Shc/p46Shc forward 5′-GGCCCTGGACATGAACAA-3′ and reverse 5′-CCATGACTTTGTCGTTGGGA-3′; p66Shc forward 5′-CTGAAACTGTCTGGGTCTGAG-3′ and reverse 5′-TAGCCTGGTTGGACCTCT-3′; β-actin control primers were provided with the TissueScan kit and were used as a reference gene.

### RNA-seq

Total RNA was extracted by Trizol from whole tumors that were excised from WT (*n* = 5), p52SHC-KO (*n* = 5), and the p66SHC-KO (*n* = 5), followed by library preparation using Illumina’s TruSeq RNA library kit and sequencing on an Illumina HiSeq2500 (Illumina, Inc., San Diego, CA). Individual libraries were prepared for each sample, indexed for multiplexing, and then sequenced on an Illumina HiSeq2500. The Trim Galore program (v0.4.1) was used to trim bases with a Phred quality score < 20 [https://www.bioinformatics.babraham.ac.uk/projects/trim_galore/] and only reads with a Phred quality score equal or higher than 20 were taken for analysis. The RSEM program function “rsem-prepare-reference” (v1.3.0) was used to extract the transcript sequences from the rat genome (build Rnor6.0) [26] and to generate Bowtie2 indices (Bowtie2 v2.2.8) [26], followed by read alignment using the “rsem-calculate-expression” function. Differential expression analysis was performed using the Bioconductor package DESeq2 version 1.12.4 to compute log2 fold changes and false discovery rate-adjusted *P* values [27]. Statistical significance was determined at a false discovery rate threshold of 0.05. Data were analyzed for molecular and functional pathway enrichment using Ingenuity Pathway Analysis (IPA; Qiagen, Redwood City, CA, USA).

### Western blot

Frozen OCT-embedded human breast tissue (tumor and matched normal) was obtained from the Medical College of Wisconsin Tissue Bank. For these specimens, normal tissue is defined as being distant from the tumor of the same patient that has no visual tumor present as determined by the pathologist at the time of procurement. Specimens were washed with PBS to remove OCT compound before protein extraction, performed using a Potter-Elvehjem homogenizer followed by sonication in RIPA buffer. Lysates were centrifuged to remove debris, and protein was measured using the BCA assay (Thermo Fisher) and frozen at − 80 °C before SDS-PAGE western blot analysis. SHC protein expression was analyzed using anti-SHC1 antibody (EMD Millipore 06-203) (dilution 1:2000) followed by IRDye800 secondary antibodies (Li-COR). Total protein was assessed by REVERT (Li-COR). Human INSTA-Blot™ Breast Tissue OncoPair (Novus Biologicals #NBP2-30128) ready-to-use PVDF membranes were used to assess total SHC proteins expressions in human breast cancer and normal adjacent tissue, 14 μg total protein per sample. Total protein was assessed by amido black stain provided by the manufacturer. Membrane was blotted with anti-SHC1 supplemented with anti-p66SHC antibody (Biosource 44829M) (dilution 1:1000) followed by goat anti-rabbit-HRP and goat anti-mouse-HRP (Biorad) (dilution 1:2000), according to manufacturer’s protocol. For rats, tumor and distant normal mammary samples from the same rat were also compared. Thirty micrograms of total protein was electrophoresed and transferred to Immobilon-FL membrane (Millipore). Membranes were blocked in Odyssey Blocking Buffer/TBST 1:1 and then blotted with anti-SHC1 antibody (EMD Millipore) (dilution 1:1000) and anti-β-actin (Sigma) (dilution 1:2000) overnight in 1:1 Odyssey Blocking Buffer/TBST. Membranes were rinsed in TBST and incubated with appropriate IRDye secondary antibodies (Li-COR) at dilution 1:15,000. Membranes were scanned on a LI-COR Odyssey scanner.

### Immunohistochemistry

Staining for ER, PR, HER2, and SHC1 was performed using Dako EnVision FLEX mini Kit on a Dako Autostainer Omnis (Agilent, Santa Clara, CA). High-resolution digital images were captured at × 20 using a Pannoramic 250 Flash III slide scanner (3DHISTECH Ltd., Budapest, Hungary). ER, PR, HER2, and SHC1 were detected using EnVision FLEX Target Retrieval Solution Low pH (Citrate buffer, pH 6.1, Agilent) and incubation with anti-ER (1:200, ab3575, Abcam, Cambridge, MA), anti-PR (1: 2000, ab16661, Abcam), anti-HER2 (1:400, A0485, Agilent), and anti-SHC (1:50, 06-203, Sigma) for 30 min, followed by secondary antibody (Polymer) for 30 min. Counterstaining with hematoxylin, dehydration, clearing, and coverslipping was performed. The Dako HER2 antibody recognizes rat HER2 [28].

### Statistical analysis

Tumor latency and multiplicity between genotypes were analyzed by a multiple comparison test using Tukey’s method and adjusted using single-step Bonferroni. To compare tumor latency at 5, 10, and 15 weeks, a nonparametric survival regression model was used (with tumors never developed being treated as right-censored observations), while pair-wise comparisons were performed using a two-sample melded BPCP test. Total tumor burden was calculated as the sum of all tumors extracted from each rat. Total tumor burden between genotypes was compared using the non-parametric Kruskal-Wallis test (since the data appeared to be non-normal), followed by testing of pairwise contrasts using Dunn’s test. Multiplicity adjustments for the *P* values were done using Holm’s method. For western blot semi-quantitative analysis, group means were compared using the Mann-Whitney rank sum test.

## Results

### Expression of *SHC1* gene products are upregulated in mammary tumors

**Fig. 1:**
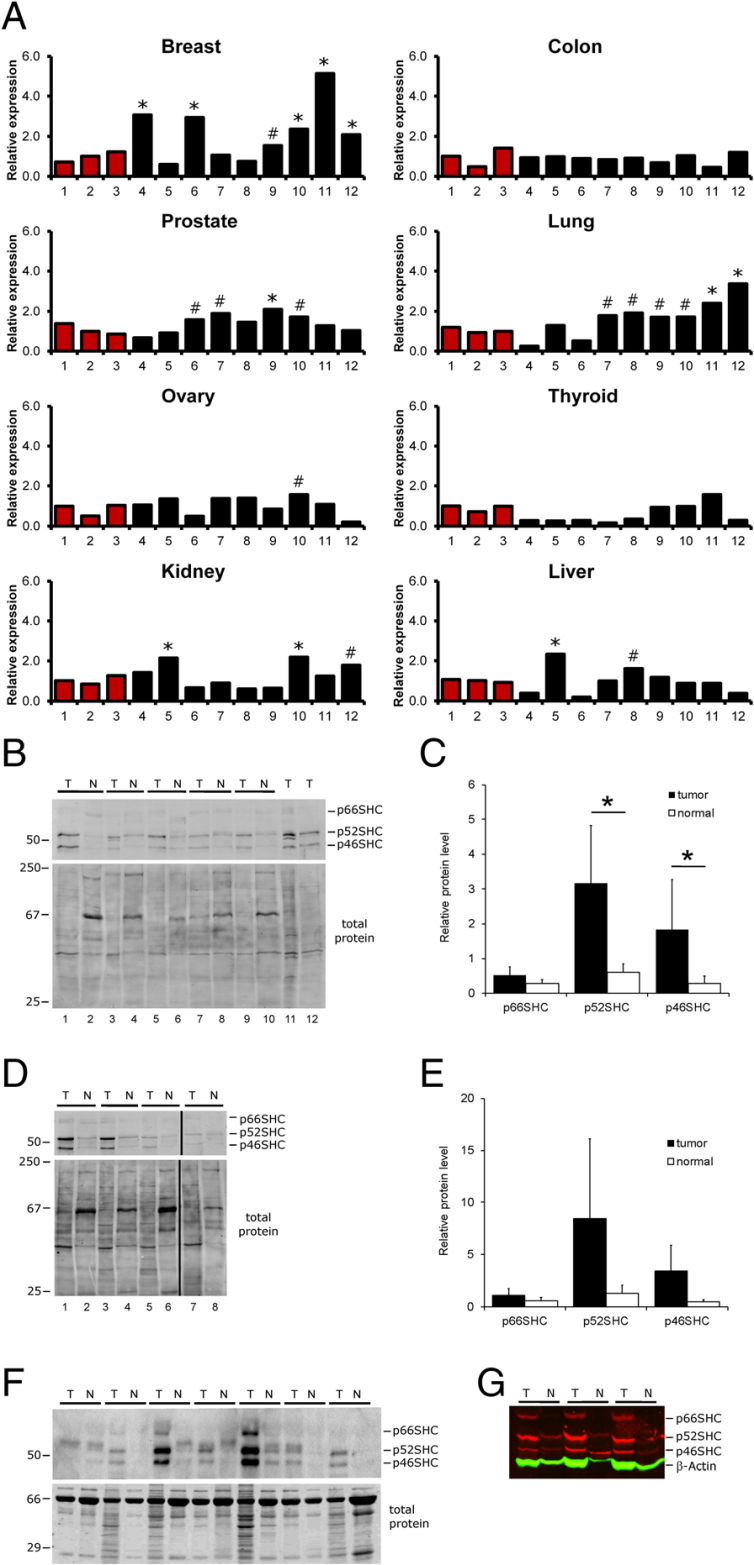
Upregulation of *SHC1* p46SHC/p52SHC transcript and SHC proteins in human breast cancers. **a** Quantitative real-time RT-PCR analysis of *SHC1* p52SHC/p46SHC transcript levels on a TissueScan cancer survey panel of the breast, colon, prostate, lung, ovary, thyroid, kidney, and liver non-tumor tissue (red bars) and tumor tissue (black bars). Expression is normalized to the median non-cancerous tissue samples. Expression of p52SHC/p46SHC transcript greater than 1.5 is indicated with a number sign, while expression greater than 2 times is indicated with an asterisk. **b** Western blot analysis of SHC1 isoform expression of paired ER+/PR+/HER2− tumor (T) and non-tumor (N) samples, 5 μg/lane. Lane numbers are indicated at the bottom. **c** Quantification of data from **b** panel by normalization to total protein, where *P = 0.008. **d** Western blot analysis of SHC1 in triple negative ER−/PR−/HER2− paired samples, 10 μg/lane. Lane numbers are indicated at the bottom. **e** Quantification of data from **d** panel by normalization to total protein. **f** INSTA-Blot Breast Tissue OncoPair membrane blotted with both c-terminal total SHC1 and n-terminal p66SHC-specific antibodies. Membranes contain seven paired tumor (T) or non-tumor (N) samples. Locations of three SHC isoforms are indicated. **g** Western blot analysis of SHC isoform expression (red bands) and β-actin (green) as a loading control in paired normal (N) and DMBA tumor (T) samples from the same rats (*n* = 3 rats)

According to the Human Protein Atlas, in breast carcinomas, there is a difference in expected outcome between patients expressing high levels of *SHC1* RNA and patients expressing low levels of *SHC1* RNA based on best separation (*P* = 0.0412), but not median separation (*P* = 0.0928) [29]. Even though these data suggest the significance of high levels of *SHC1* RNA for the worst expected outcome of breast cancer, they do not address the impact of individual protein isoforms in the progression of breast carcinomas. In mammals, *SHC1* is expressed as two distinct transcripts based on the utilization of alternate promoters [9]. The shorter (3062 bp) transcript, termed here the p52SHC/p46SHC transcript, contains in-frame initiation codons for both p52SHC and p46SHC. The longer (3481 bp) transcript, termed the p66SHC transcript, contains initiation codons for all three protein isoforms, but is considered to serve predominately as a source of p66SHC. A broad human TissueScan cancer survey panel was screened for expression of both SHC1 transcripts. Each panel contained cDNA, normalized for β-actin expression, from 9 cancer samples from individual patients of increasing stage from 8 different cancer types and three normal tissues [30]. Expression of p52SHC/p46SHC transcript was upregulated more than 2-fold (*) compared to non-cancer tissue in 5 out of the 9 breast cancer samples, with one additional sample being greater than 1.5 times (#) higher (Fig. 1a) when compared to the median non-tumor samples. According to supplied information from OriGene, most of the tumors assayed are estrogen receptor (ER) and prolactin receptor (PR) positive, with mixed Her2 positive staining (Additional file 1: Figure S1). In contrast, no significant changes in p52SHC/p46SHC transcript were observed in colon, ovary, and thyroid cancers. Prostate, lung, kidney, and liver carcinomas exhibited variable expression in p52SHC/p46SHC expression, ranging between 1.5- and 2-fold increase over normal tissue (Fig. 1a). Breast carcinomas were unique in that most samples expressed the p52SHC/p46SHC transcript 2-fold higher than the normal breast tissues. Expression of the p66SHC transcript, the lesser-expressed *Shc1* transcript, was below detectable levels in most normal and some breast tumor samples (data not shown). Additionally, p66SHC transcript levels were below detectable levels in many of the tumor samples across all cancer types (data not shown).

**Fig. 2:**
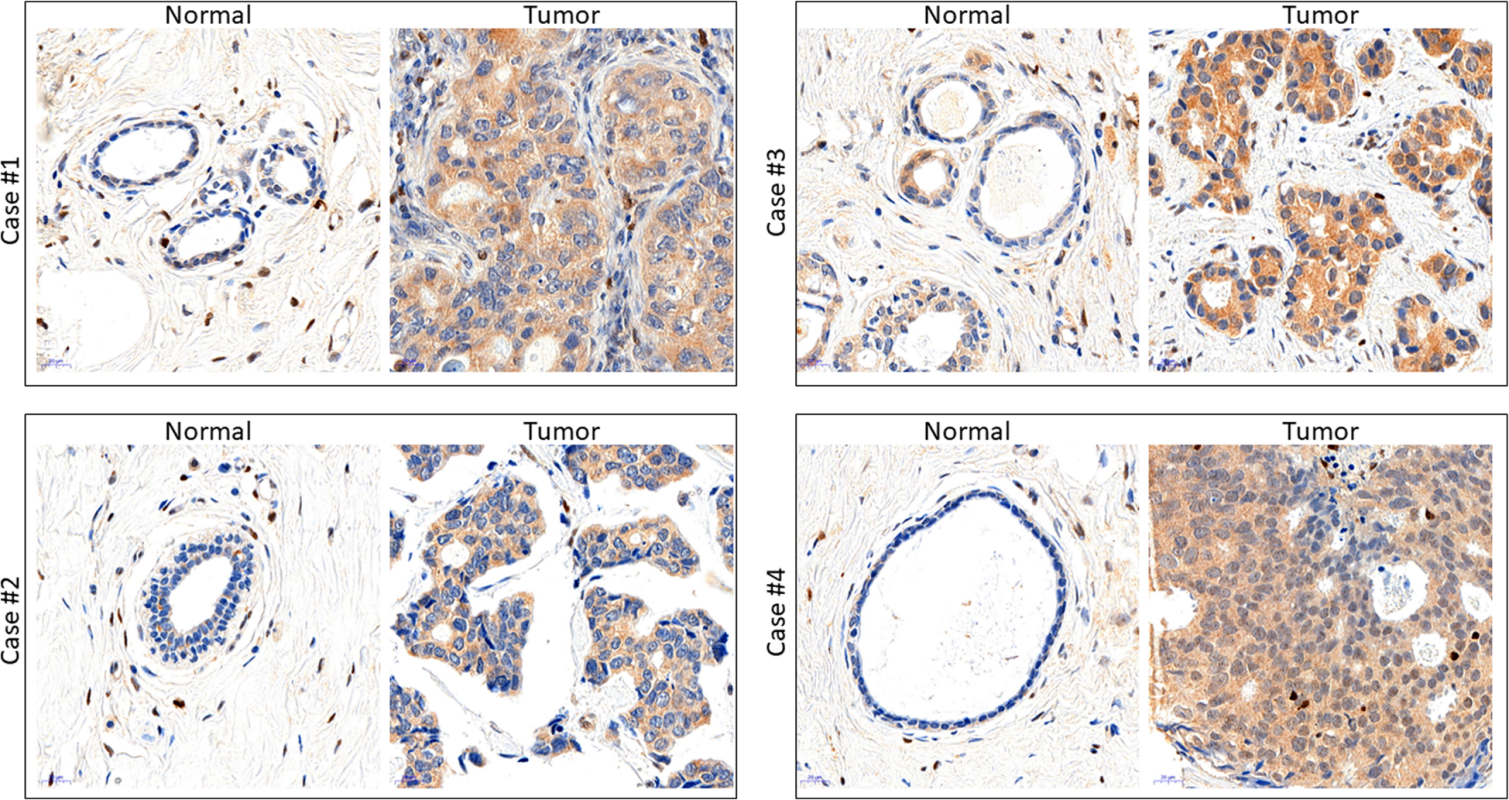
Immunohistochemistry of SHC1 proteins shows the increased staining of SHC1 proteins in breast cancer cells compared to adjacent normal breast epithelia or stromal cells in human breast cancer. There are shown 4 cases of normal tissues and 4 cases of breast cancers. Each image represents one case

To assess differences in expression of all three SHC1 protein isoforms in human breast tumors, we have analyzed 9 independent breast cancer tumor samples (T) paired with matched normal protein samples (N) from tissues obtained from MCW Tissue Bank. It is of note, that in all (five) ER+/PR+/HER2− breast cancer samples, we detected the increased expression of p52SHC and p46SHC whereas the difference in p66SHC expression was not observed (Fig. 1b, c). The tumor/stroma composition (contribution of normal mammary epithelial) was quantified by a pathologist (Additional file 5: Table S1). Although triple negative tumors appeared to have increased SHC1 proteins levels, the differences did not reach significance compared to normal tissues when normalized to the total amount of protein (Fig. 1d, e). Additionally, commercial INSTA-Blot™ OncoPair membranes were blotted with total SHC1 antibody that recognizes all three isoforms, concurrently with an N-terminal p66Shc antibody. Comparison of seven independent tumor samples of increasing grade and stage [31] with matched normal protein samples from adjacent tissues indicated that p52SHC and p46SHC isoforms were upregulated in 6 out of 7 tumor samples compared to normal-paired samples (Fig. 1f). Likewise, 3 out of 7 tumor samples expressed increased levels of p66SHC protein compared with the normal-paired breast tissues. Similar to observations made with human breast tissue, the analysis of DMBA-induced rat tumors compared with normal rat mammary tissue revealed increased expression of all SHC1 isoforms (Fig. 1g). Immunohistochemical staining of SHC1 proteins showed markedly elevated expression of SHC1 proteins in breast cancer cells compared to adjacent normal breast epithelia or stromal cells, both in human (Fig. 2) and rat tumors (Additional file 2: Figure S2).

### The p52SHC isoform is required for DMBA-induced mammary tumor formation

**Fig. 3:**
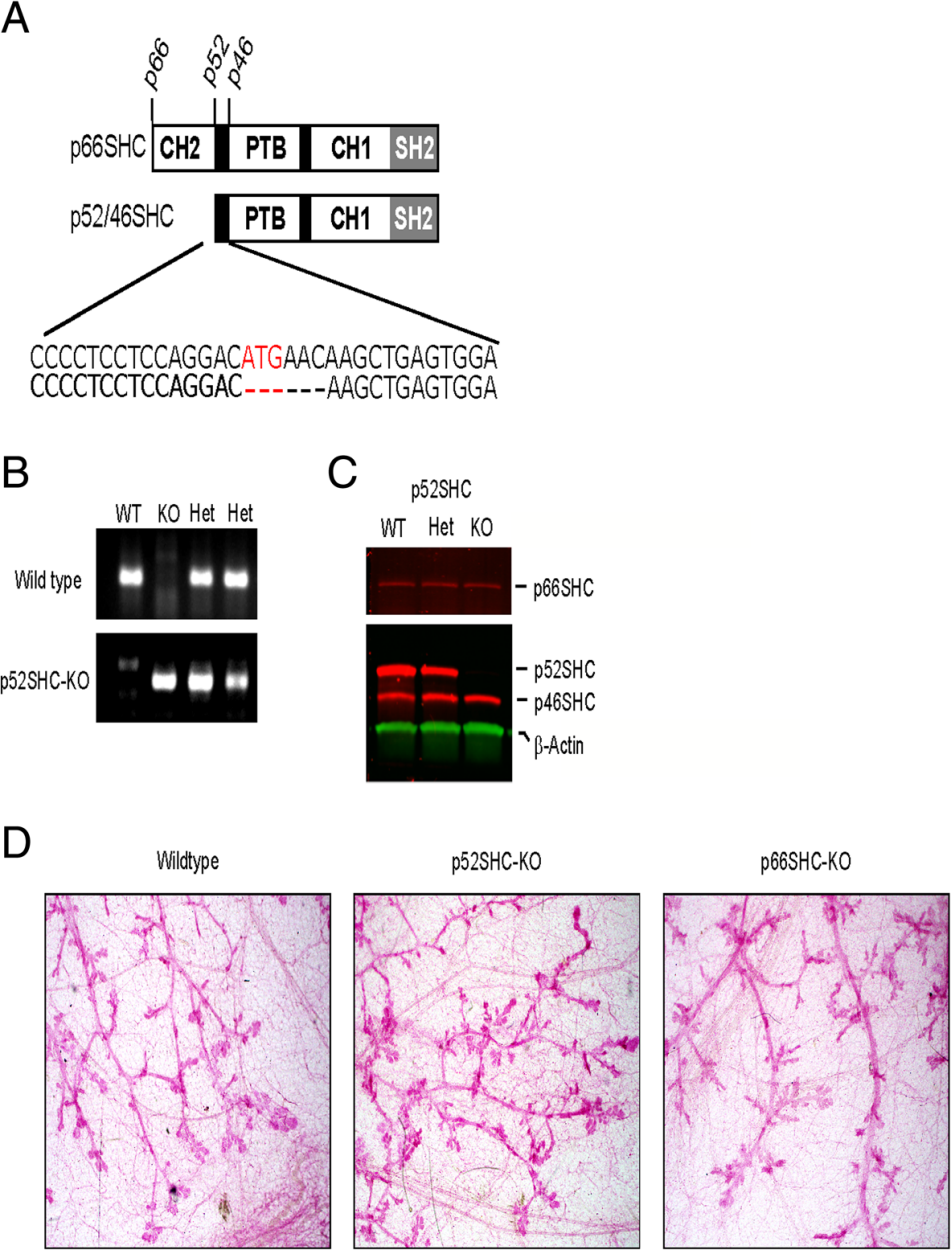
Knockout of *p52SHC* by CRISPR/Cas-9. **a** Diagrammatic representation of protein domains and ATG initiation codons of SHC isoforms that arise from the two *SHC1* transcripts. To knock out the p52SHC isoform, the initiation of the codon for p52SHC was targeted using the CRISPR/Cas-9 gene editing in the salt-sensitive (SS) rat. One founder generated a 6-base pair deletion (red) in which the p52SHC ATG was deleted. **b** PCR genotyping results of WT, p52SHC-KO, and heterozygous (Het) animals. The upper gel shows PCR reaction for WT allele and the lower gel shows PCR reaction for the mutant allele. **c** Western blot from spleen tissue showing expression of SHC isoforms (red) and β-actin (green) as a loading control in WT, heterozygous (Het), and p52SHC-KO knockout (KO) rats. **d** Carmine alum stain of the normal adult mammary epithelium of wild-type, p52SHC-KO, and p66SHC-KO rats. Magnification × 4

**Fig. 4:**
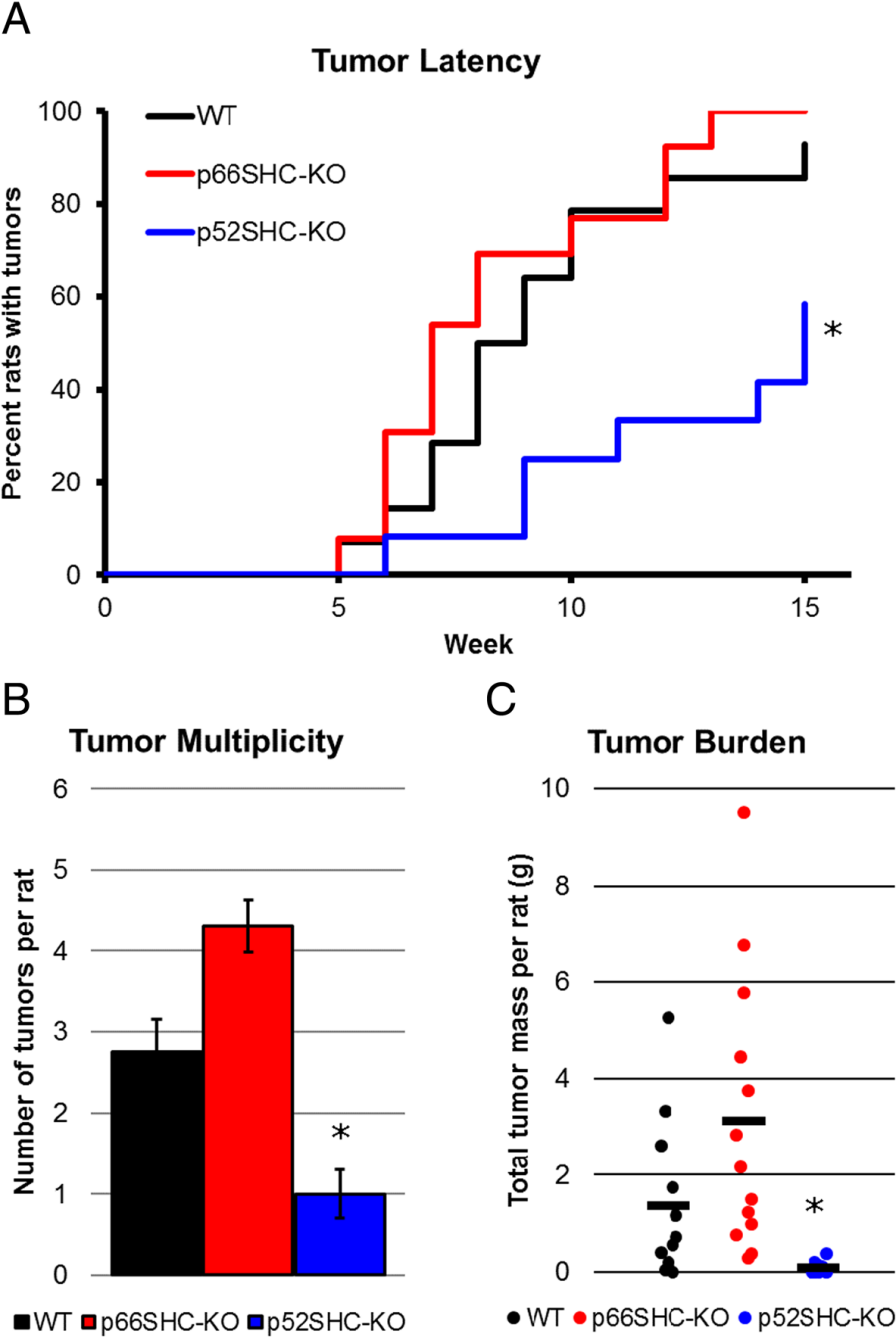
Increased tumor latency and decreased multiplicity in DMBA-induced p52SHC knockout rats. **a** Tumor latency was compared between WT (black, *n* = 14), p66SHC-KO (red, *n* = 13), and p52SHC-KO (blue, *n* = 12) DMBA-induced rats over 15 weeks. *P < 0.015. **b** The average multiplicity of tumors at 15 weeks for each genotype. Error bars represent standard error of means. *P < 0.004. **c** Total tumor burden, the sum total of mammary tumors per rat, isolated for each animal. The line indicates average tumor mass for each genotype. *P < 0.02

Individual contributions of p66SHC and p52SHC in the development and progression of breast cancer are unclear. Since both p66SHC and p52SHC protein levels were elevated in some human and in rat DMBA-induced tumors (Fig. 1d, e), we utilized rats in which p66SHC or p52SHC proteins were genetically removed to study the consequence of their loss on DMBA-induced mammary tumor formation. The p66SHC knockout (p66SHC-KO) was generated by targeting the unique sequence of p66Shc using Zinc Finger technologies and has been previously described [19]. For the first time in any species, a p52SHC knockout was generated using CRISPR/Cas-9 genomic editing [32]. A single founder was generated in which 6-base pairs, including the p52SHC ATG initiation codon, were deleted (termed p52SHC-KO, Fig. 3a). This deletion resulted in a 2-amino acid loss from p66SHC protein that was not predicted to alter p66SHC function. The deleted amino acids are not located in any known functional protein domains [12]. Furthermore, p52SHC-KO rats do not exhibit common phenotypes associated with p66SHC loss of function when placed on a high salt diet (data not shown), suggesting a normal p66SHC function in the p52SHC-KO rats. Additionally, the p46SHC sequence was preserved. Importantly, the mutation did not alter expression of either p66SHC or p46SHC protein levels, while p52SHC isoform lacks completely as detected by PCR genotyping (Fig. 3b) and western blot using a total SHC1 antibody (Fig. 3c, Additional file 3: Figure S3). Absence of p66SHC band by western blot analysis in p66SHC-KO, but not in WT or p52SHC-KO rats is also demonstrated (Additional file 3: Figure S3). Additionally, both p52SHC-KO and p66SHC-KO rats can be maintained at homozygous knockouts and give birth to average litter size and pup size (data not shown). There was no difference in the mammary gland structure between WT, p52SHC-KO, and p66SHC-KO rats (Fig. 3d). No masses, cysts or other lesions were identified across all three groups. Thus, changes in the development of mammary glands due to p52SHC or p66SHC ablation are likely not responsible for differences in DMBA-induced mammary tumor formation. Following administration of DMBA, loss of p52SHC resulted in an increase in tumor latency and a decrease in tumor incidence to 58.3% (7 of 12) by 15 weeks, compared to WT (92.9%, 13 of 14) and p66SHC-KO (100.0%, 13 of 13), (*P* = 0.038 and *P* = 0.003, respectively, Fig. 4a). Differences in tumor development were apparent as early as 10 weeks post-DMBA in a pair-wise comparison, 25.0% (3 of 12) p52SHC-KO rats had tumors compared to 78.6% (11 of 14) for WT and 76.9% (10 of 14) p66SHC-KO (*P* = 0.02 and *P* = 0.03 respectively). There was no difference in latency between WT and p66SHC-KO at any point during the 15-week time course (*P* = 0.4). During the 15 weeks that tumor progression was analyzed, rats were individually palpated for the presence of tumors. It may be possible that very small lesions were missed in the analysis. In addition to increased latency, p52SHC-KO rats also had lower tumor multiplicity, averaging 1.0 ± 0.3 tumors per rat compared to 2.75 ± 0.4 and 4.31 ± 0.3 tumors in WT and p66SHC-KO, respectively (*P* = 0.002 and *P* < 10^−4^, Fig. 4b). Furthermore, p52SHC-KO rats had a lower overall tumor burden per rat, 0.08 ± 0.04 g, compared to WT, 1.34 ± 0.5 g, and p66SHC-KO rats, 3.10 ± 0.8 g, respectively (*P* < 0.02 and *P* < 10^−5^, Fig. 4c). In contrast, p66SHC-KO rats trended towards increased multiplicity and tumor burden compared to WT DMBA-induced rats but was not significant (*P* = 0.09 and *P* = 0.08). We have carried out immunostaining for ER, PR, and HER2 of DMBA-induced mammary tumors formed in our genetically modified rats (Additional file 6: Table S2). All analyzed DMBA-induced tumors were ER and PR positive and HER2 negative.

### RNA-seq analysis reveals significant changes in the p52SHC tumor transcriptome

**Fig. 5:**
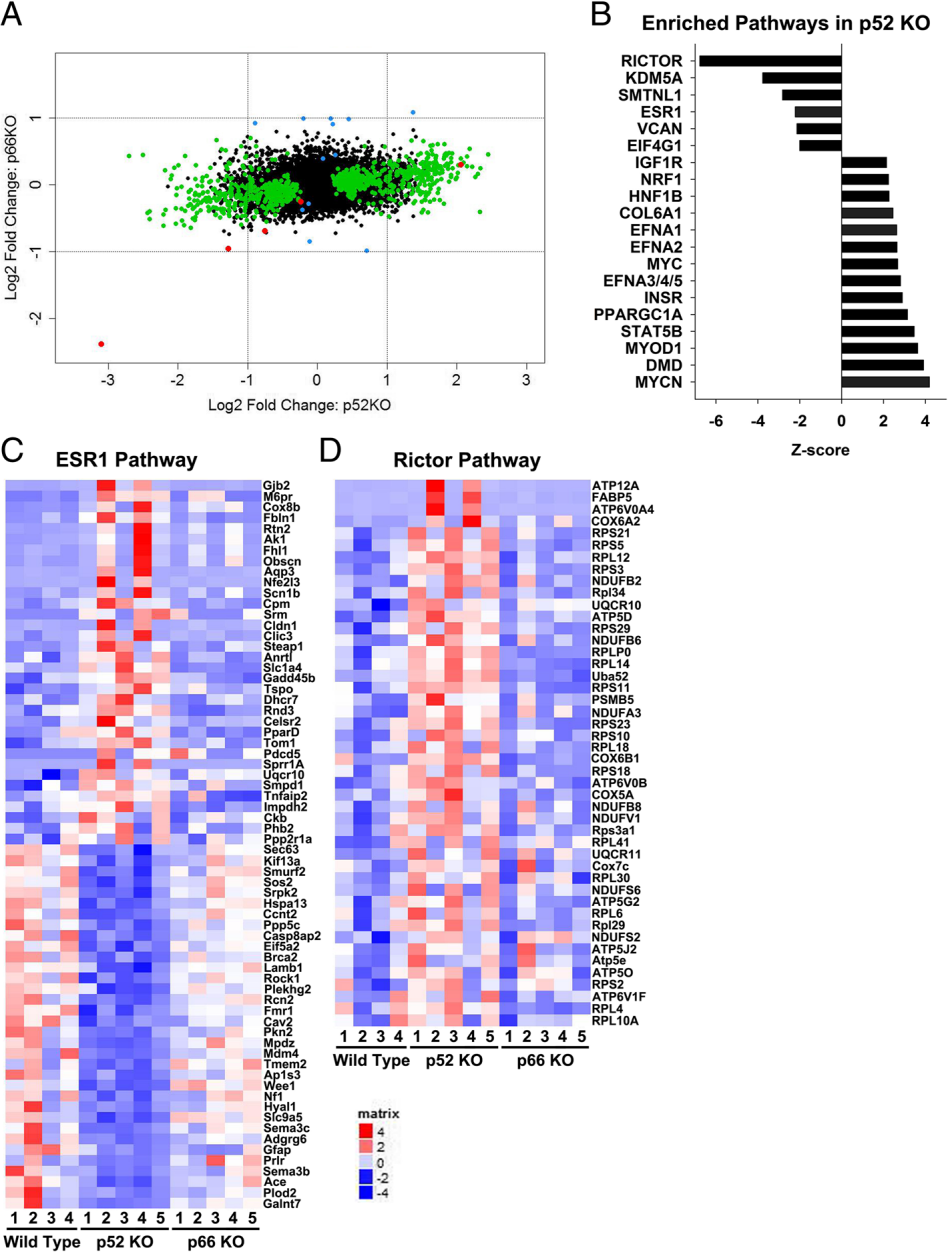
RNAseq analysis reveals enriched genes dis-regulated by loss of p52SHC. **a** Histogram comparing log2 fold change in transcript expression in p52SHC-KO tumors compared to WT (*X*-axis) with fold change in transcript expression in p66SHC-KO tumors (*Y*-axis). Green dots symbolize genes that are differentially expressed in only one knockout group. Blue dots symbolize genes that are differentially expressed in p52SHC-KO and p66SHC-KO tumors in the opposite directions. Red dots symbolize differentially expressed genes p52SHC-KO and p66SHC-KO tumors in the same direction. **b** Bar graph indicating the top 20 enriched pathways with *Z*-score greater than ± 2 in p52SHC-KO tumors, ranked by *Z*-score. **c**–**e** Heat map of ESR1 (**c**) and RICTOR (**d**) pathway targets identified by RNAseq analysis of wild-type, p52SHC-KO, and p66SHC-KO DMBA-induced tumors

To begin identifying gene networks that are mediated by p52SHC and p66SHC in breast cancer, RNA-seq was performed on mammary tumors derived from WT (*n* = 4), p52SHC-KO (*n* = 5), and p66SHC-KO (*n* = 5) rats. Compared with the WT tumors, 893 genes were differentially expressed (DE; FDR < 0.05) in the p52SHC-KO tumors, whereas only 18 DE genes were detected in the p66SHC-KO tumors (Fig. 5a, Additional file 7: Table S3). Although the WT and p66SHC-KO tumors were largely indistinguishable, gene ontology (GO) enrichment analysis using IPA revealed multiple enriched GOs in p52SHC-KO tumors compared with WT (Fig. 5b), including the ESR1 pathway (68 DE genes, *Z*-score = − 2.2, *P* = 1.46 × 10^−3^) and the RICTOR/mTORC2 pathway (46 DE genes, *Z*-score = − 6.8, *P* = 6.17 × 10^−19^). Collectively, these data provide the first in vivo link between p52SHC isoform and well-known drivers of breast cancer.

## Discussion

Our data that p52SHC-KO rats have increased latency and decreased tumor multiplicity suggest that delayed onset of tumorigenesis observed in the mouse model of polyomavirus middle T antigen (MT)-induced mammary tumors in which critical tyrosine phosphorylation residues in SHCA are mutated (termed *ShcA*^*3F*^ and *ShcA*^*2F*^) resulting in fewer tumors are likely due to phosphorylation defects of the p52SHC isoform [17]. Indeed, the same phosphorylation of p66SHC can antagonize downstream RTK signaling, whereas phosphorylated p52SHC activate signaling downstream of RTK [11]. In the p52SHC-KO animals, we report that despite being derived from the same mRNA transcript, those p46SHC protein levels are not increased as may be expected. This is likely due to the role p46SHC is performing in normal cells. Recent data suggests that p46SHC expression is restricted to mitochondria where it acts to tightly regulate thiolase and lipid oxidation [13]. As such protein levels are likely to be highly regulated. However, in cancer cells, where p52SHC and p46SHC are overexpressed, studies have indicated p46SHC is able to co-precipitate (along with p52SHC, and sometimes p66SHC) with key factors regulating tumorigenesis and proliferation and are unable to distinguish distinct functions of p46SHC or p52SHC [15, 16, 18, 33–36]. Data presented here (Fig. 4) is the first to show that p52SHC is the predominant *Shc1* isoform that mediates mammary tumorigenesis and that p66SHC and p46SHC are less likely to influence tumorigenesis to a major extent. In p52SHC-KO animals, even partial redundancy of either p66SHC or p46SHC may explain why some tumors do form. Furthermore, it must be mentioned that MT-induced mammary tumor model is a non-random model of breast cancer, in which MT interacts and activates a panel of host signaling molecules components of ERK, AKT and other signaling pathways, including SHC/MAPK cascade [37]. Since DMBA-induced mammary tumors in rats represent a model of random mutagenesis it resembles naturally occurring human ER+/PR+ luminal breast tumors [25, 38]. Evidence suggests that p66SHC, while expendable in DMBA-induced tumors (Fig. 4), may function in tumors with higher metastatic potential [5–7], and as such p66SHC-KO rats should be used to study more metastatic models of breast cancer.

Estrogen signaling through ESR1, also known as ERα, is well known in human breast cancers. Several lines of evidence suggest co-regulation of 17β-estradiol (E2, the ligand for ERα and ERβ) and growth factor signaling, such as IGF and EGF, in the traditional genomic regulation of gene transcription [4]. In addition to serving as a co-regulator mediating growth factor signaling with the canonical nuclear action of ERα, recent works have highlighted non-genomic functions of ERα in reciprocal activation of growth factor signaling pathways [4, 39]. Non-genomic activation of membrane ERα has been identified in tumor cells that become hypersensitive to E2 during acquired resistance to hormone deprivation therapies, which appears to be dependent on SHC1 proteins. For example, SHC1 proteins directly interact with ERα and mediate its translocation to the cell membrane for docking with IGF-1R, which subsequently leads to activation of the RAS/MAPK signaling cascade at the plasma membrane [40–42]. Ultimately, the SHC1-mediated crosstalk between ERα and IGF-1R leads to robust activation of proliferation and suppression of apoptosis [40–42]. However, despite the evidence that SHC1 is a key mediator of non-genomic ERα mechanisms of resistant to anti-estrogen therapy, the functionally significant role(s) of SHC1 isoforms were largely unclear. We addressed this question using RNAseq analysis of the p52SHC-KO and p66SHC-KO tumors, which demonstrated that loss of p52SHC disrupts ERα signaling, whereas deletion of p66SHC had no effects (Fig. 5c). Together, these data suggest that genes identified in our RNA-seq analysis of p52SHC-KO tumors represent a partial list of genes that are regulated by non-genomic ERα activation of MAP Kinase/ERK signaling pathways in a p52SHC-dependent manner. Analysis of ERK activation in primary mammary epithelial cells isolated from WT and p52ShcKO rats revealed a slight decrease in ERK activation in p52ShcKO estradiol-treated cells when compared to cells isolated from WT (Additional file 4: Figure S4).

Identification of the RICTOR signaling pathway as being significantly downregulated in p52SHC-KO tumors signifies a potential novel interaction between p52SHC and RICTOR/mTORC2 signaling pathway in mammary carcinogenesis. RICTOR, a defining component of mTORC2 (mammalian target of rapamycin complex 2), is involved in regulating kinases of the AGC group (named after the protein kinases A, G, and C families [43]. Majority of the RICTOR targets identified as being differentially regulated (Fig. 5d) represent genes identified in a microarray of liver-specific conditional RICTOR knockout mice as components of a ribosomal protein complex (EIF signaling) or oxidative phosphorylation pathways negatively regulated by RICTOR/mTORC2 signaling [44]. Coincidently, EIF2 signaling and oxidative phosphorylation represent two canonical pathways significantly dysregulated in p52SHC-KO mammary tumors (*P* value 2.18 × 10^−12^ and 2.18 × 10^−10^, respectively; data not shown). While mTORC2-dependent and mTORC2-independent functions of RICTOR are known [45–48], genes identified in the RNA-seq analysis appear to represent mTORC2-dependent functions of RICTOR signaling pathways, as loss of mTOR itself also causes significant changes in oxidative phosphorylation and mitochondrial dysfunction [49, 50]. Additionally, mTOR is reported to functionally co-regulate RNA polymerase II-dependent genes, such as ribosomal proteins, many of which are also identified in our RNA-seq analysis [51]. These data suggest a previously unrecognized role for p52SHC in the regulation of RICTOR/mTORC2 signaling pathways.

## Conclusion

In summary, our data strongly suggest that p52SHC-dependent signaling is required for mammary tumor progression and suggest p52SHC may serve as node interlinking several critical signaling pathways implicated breast cancer progression. Lack of overlap of genes identified as being dysregulated in ESR1 and RICTOR, signaling suggest p52SHC serves distinct functions regulating each of these pathways. Each of these pathways has been targeted therapeutically in order to combat the progression of mammary cancers in women. Our finding that p52SHC appears to be a central hub regulating these pathways provides a rationale for an exciting target for new drug therapies.

## Additional files

Additional file 1: **Figure S1.** Hormone receptor expression status of human breast cancer samples used for qRT-PCR. Quantitative real-time RT-PCR of p52Shc/p46Shc transcript levels from TissueScan cancer survey panel of normal (red bars) and breast cancer (black bars), as seen in Fig. 1. Corresponding expression of Estrogen Receptor (ER), Prolactin Receptor (PR), or Her2 Receptor (Her2) status of breast cancers used in the qRT-PCR assay. NA, not applicable; +, positive staining; +w; weak positive staining; BL, borderline expression; NR, status not reported. (TIFF 621 kb)

Additional file 2: **Figure S2.** Immunohistochemistry of SHC1 proteins shows the increased staining of SHC1 proteins in mammary carcinoma cells compared to adjacent normal breast epithelia or stromal cells in DMBA-induced rat breast cancer. Shown is expression of Shc1 proteins in two wild-type rat DMBA tumors. (TIF 794 kb)

Additional file 3: Figure S3. Western blot analysis of SHC1 isoforms expression in WT and genetically modified rat spleen tissue (A, top panel), freshly isolated mammary tissue fragments (A, bottom panel) and DMBA-induced mammary tumor (B). Longer exposure was used to detect p66Shc expression (A, top panel). Brackets in B indicate that tissues are isolated from the same animal. Upper panel (B) is blotted with anti-SHC1 antibodies. Relative total protein levels (B, lower panel) were determined by REVERT Total Protein Stain (LI-COR). Samples were run on the same gel, but were not contiguous. (TIF 330 kb)

Additional file 4: **Figure S4.** Western blot analysis of ERK1/2 stimulation. Primary mammary epithelial cells isolated from wild-type or p52ShcKO rats were serum-starved overnight and stimulated for 5 min with b-estradiol at the indicated concentrations. Western blot analysis is carried out with phosphospecific ERK antibodies. Positions of phospho-ERK 1 and 2 are indicated by arrows. Shown is a representative experiment out of 3. (TIF 105 kb)

Additional file 5: **Table S1.** Tumor sample composition. Pathologist quality control report on the cellular composition of tumor specimens from MCW Tissue Bank. Necrosis was not detected in any sample. * Remaining tissue was not sufficient for analysis. (TIF 197 kb)

Additional file 6: **Table S2.** ER, PR and HER2 staining of DMBA-induced mammary tumors formed in wild-type and genetically modified rats. Staining for ER, PR and HER2 were achieved using Dako EnVision FLEX mini Kit on a Dako Autostainer Omnis (Agilent, Santa Clara, CA) and high-resolution digital images were captured at × 20 using a Pannoramic 250 Flash III slide scanner (3DHISTECH Ltd., Budapest, HUNGARY). (TIF 122 kb)

Additional file 7: **Table S3.** Transcripts differentially expressed in p66SHC-KO tumors compared to tumors from wild-type control animals. BaseMeans value WT and p66SHC-KO tumor samples (*N* = 4, *n* = 5, respectively). Listed is log2 fold change between WT and p66SHC-KO tumor and adjusted *P* value. (TIF 345 kb)
