# Supplementary figures and images for: The p52 isoform of SHC1 is a key driver of breast cancer initiation
Source: Breast Cancer Res. 2019 Jun 15;21:74. doi: 10.1186/s13058-019-1155-7 (PMC6570928; doi:10.1186/s13058-019-1155-7)

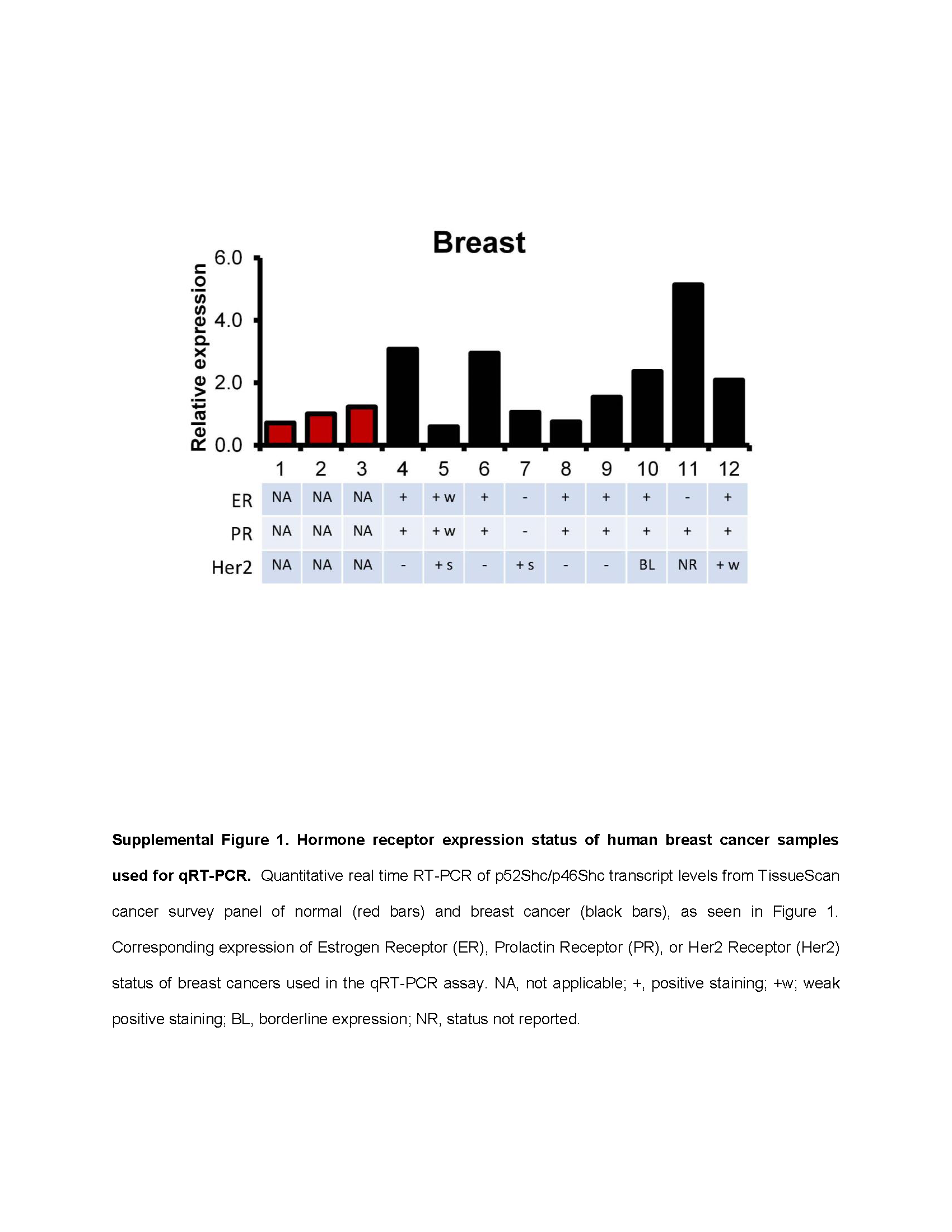

Supplement: Supplementary file 1 — Figure S1. Hormone receptor expression status of human breast cancer samples used for qRT-PCR. Quantitative real-time RT-PCR of p52Shc/p46Shc transcript levels from TissueScan cancer survey panel of normal (red bars) and breast cancer (black bars), as seen in Fig. 1. Corresponding expression of Estrogen Receptor (ER), Prolactin Receptor (PR), or Her2 Receptor (Her2) status of breast cancers used in the qRT-PCR assay. NA, not applicable; +, positive staining; +w; weak positive staining; BL, borderline expression; NR, status not reported. (TIFF 621 kb) [file 13058_2019_1155_MOESM1_ESM.tiff]

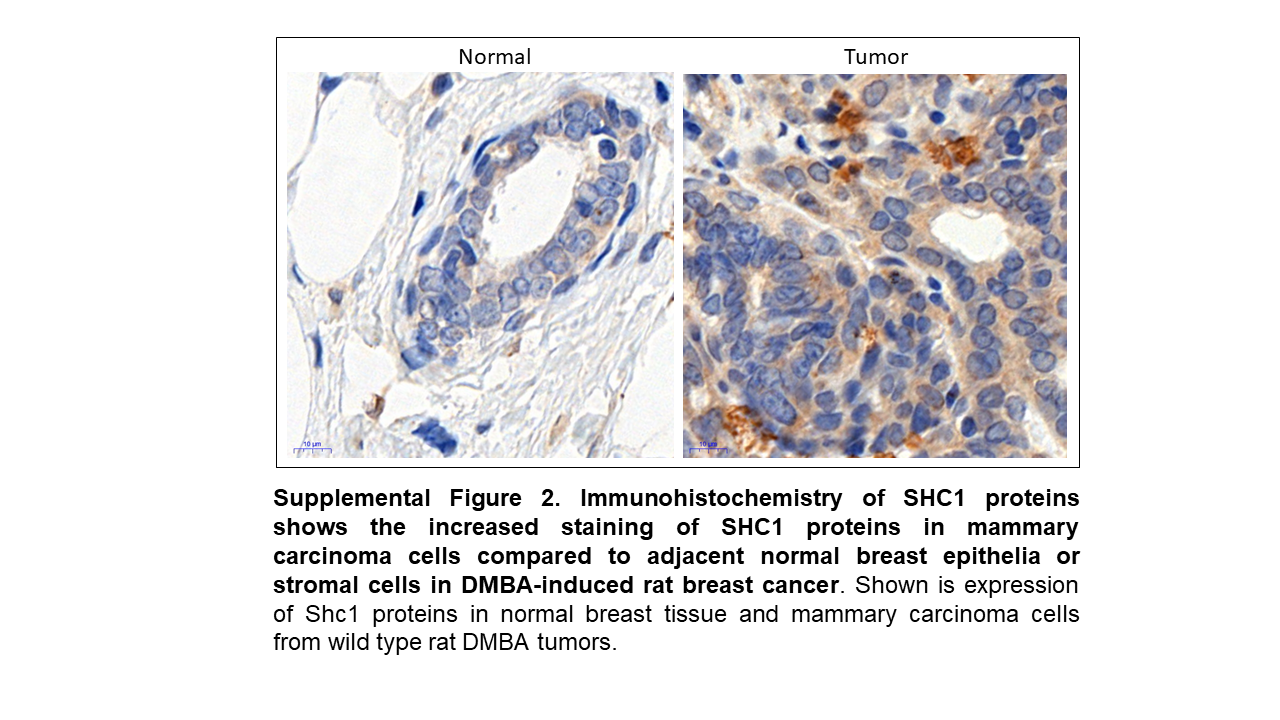

Supplement: Supplementary file 2 — Figure S2. Immunohistochemistry of SHC1 proteins shows the increased staining of SHC1 proteins in mammary carcinoma cells compared to adjacent normal breast epithelia or stromal cells in DMBA-induced rat breast cancer. Shown is expression of Shc1 proteins in two wild-type rat DMBA tumors. (TIF 794 kb) [file 13058_2019_1155_MOESM2_ESM.tif]

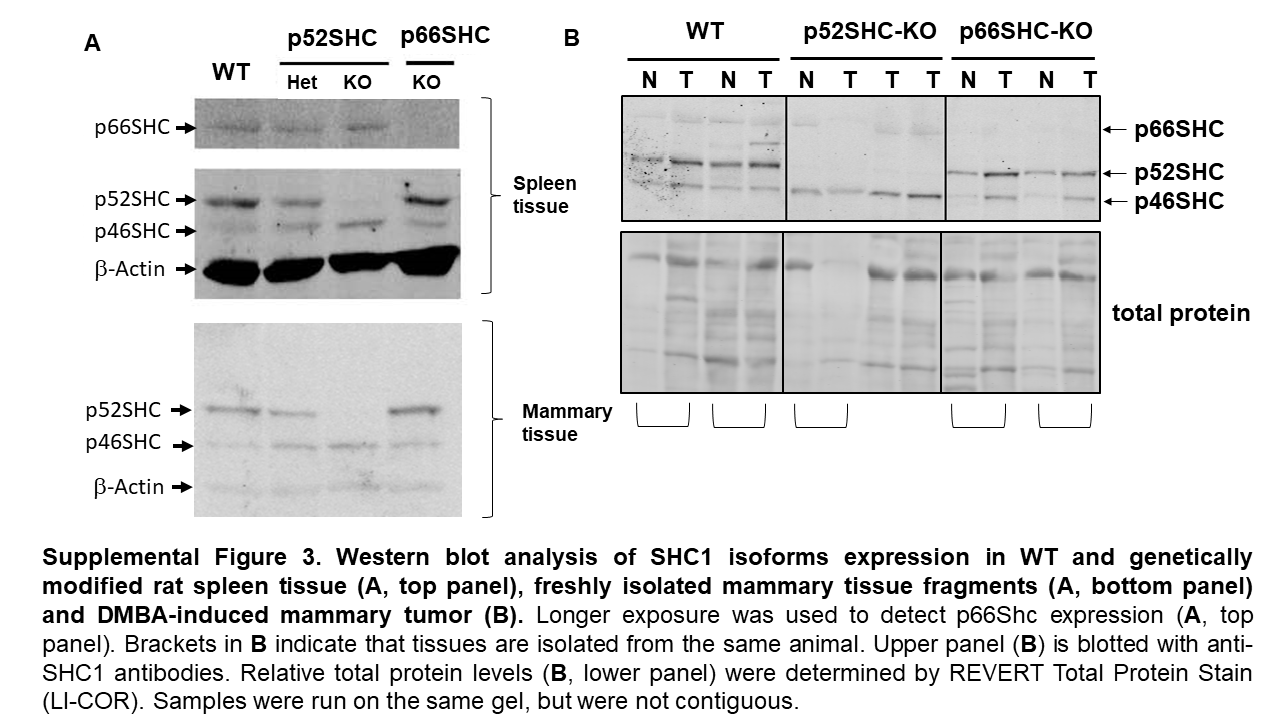

Supplement: Supplementary file 3 — Figure S3. Western blot analysis of SHC1 isoforms expression in WT and genetically modified rat spleen tissue (A, top panel), freshly isolated mammary tissue fragments (A, bottom panel) and DMBA-induced mammary tumor (B). Longer exposure was used to detect p66Shc expression (A, top panel). Brackets in B indicate that tissues are isolated from the same animal. Upper panel (B) is blotted with anti-SHC1 antibodies. Relative total protein levels (B, lower panel) were determined by REVERT Total Protein Stain (LI-COR). Samples were run on the same gel, but were not contiguous. (TIF 330 kb) [file 13058_2019_1155_MOESM3_ESM.tif]

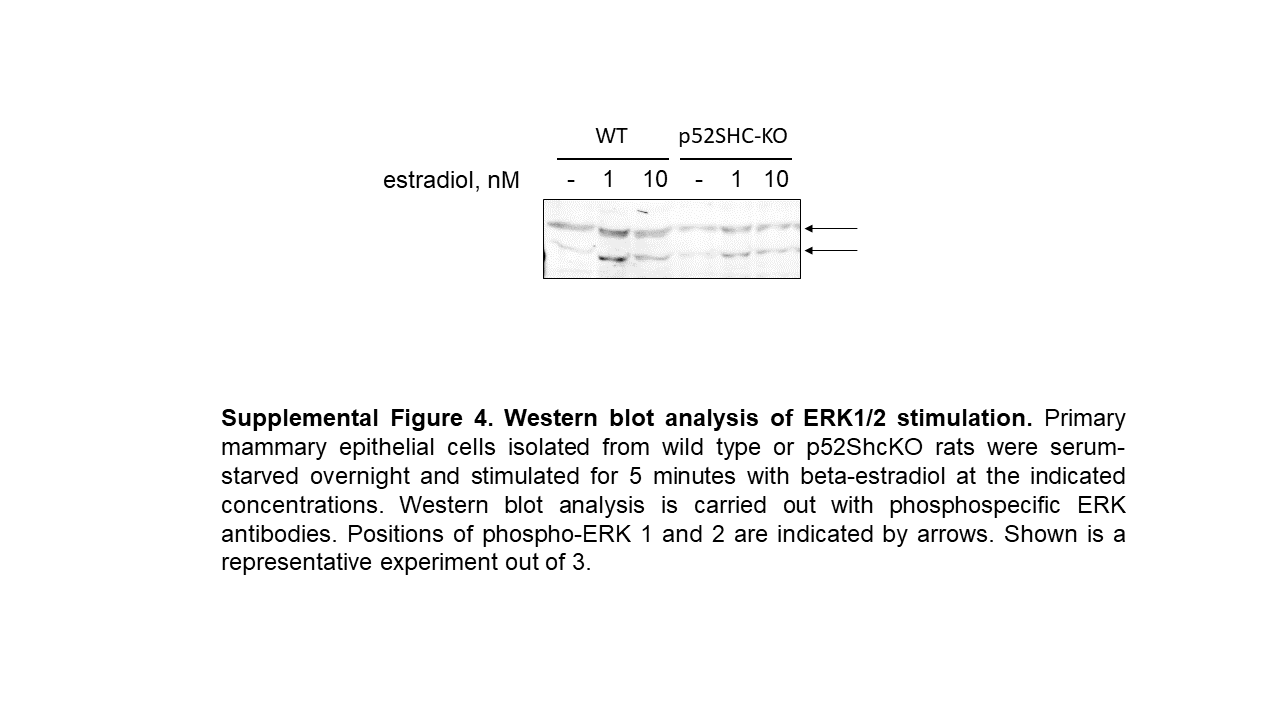

Supplement: Supplementary file 4 — Figure S4. Western blot analysis of ERK1/2 stimulation. Primary mammary epithelial cells isolated from wild-type or p52ShcKO rats were serum-starved overnight and stimulated for 5 min with b-estradiol at the indicated concentrations. Western blot analysis is carried out with phosphospecific ERK antibodies. Positions of phospho-ERK 1 and 2 are indicated by arrows. Shown is a representative experiment out of 3. (TIF 105 kb) [file 13058_2019_1155_MOESM4_ESM.tif]

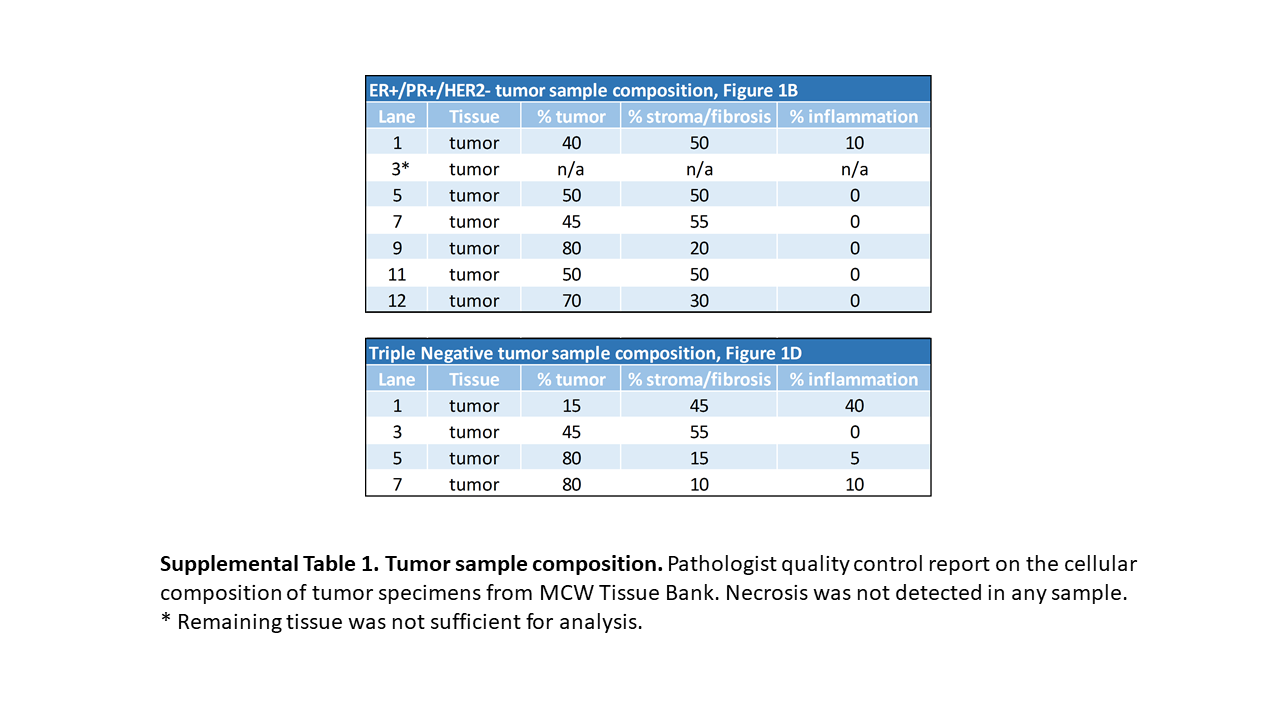

Supplement: Supplementary file 5 — Table S1. Tumor sample composition. Pathologist quality control report on the cellular composition of tumor specimens from MCW Tissue Bank. Necrosis was not detected in any sample. * Remaining tissue was not sufficient for analysis. (TIF 197 kb) [file 13058_2019_1155_MOESM5_ESM.tif]

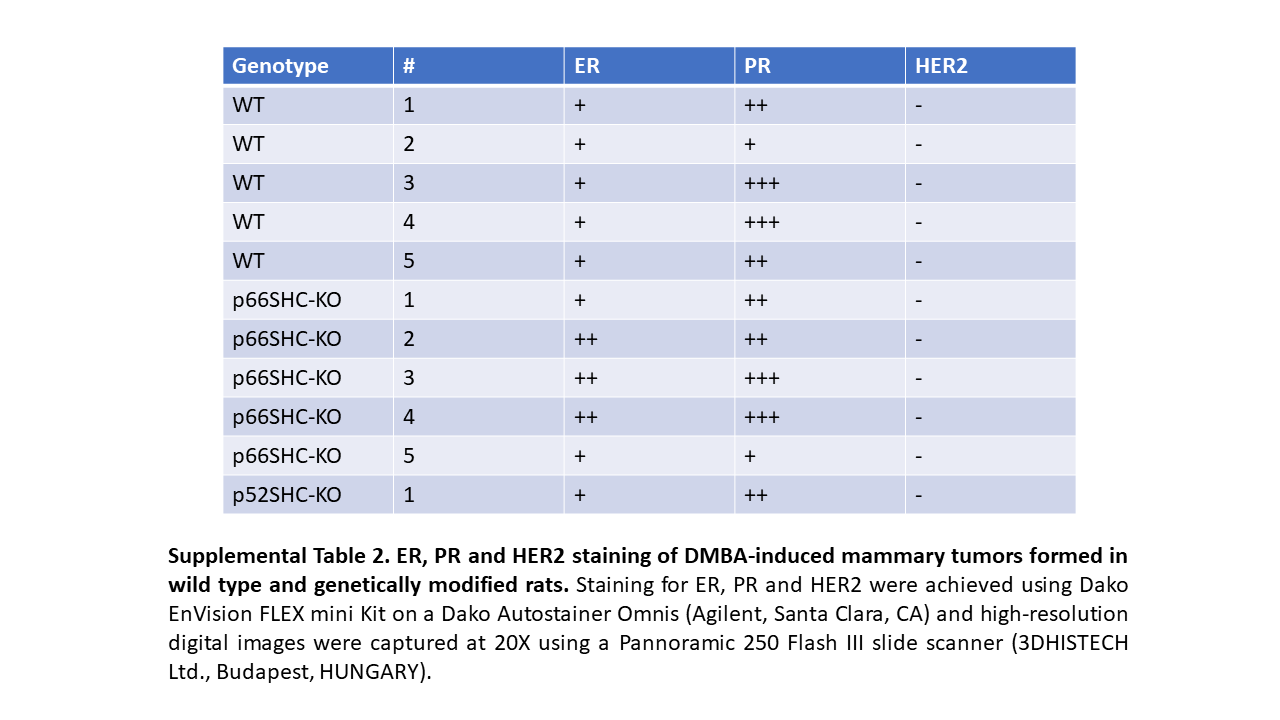

Supplement: Supplementary file 6 — Table S2. ER, PR and HER2 staining of DMBA-induced mammary tumors formed in wild-type and genetically modified rats. Staining for ER, PR and HER2 were achieved using Dako EnVision FLEX mini Kit on a Dako Autostainer Omnis (Agilent, Santa Clara, CA) and high-resolution digital images were captured at × 20 using a Pannoramic 250 Flash III slide scanner (3DHISTECH Ltd., Budapest, HUNGARY). (TIF 122 kb) [file 13058_2019_1155_MOESM6_ESM.tif]

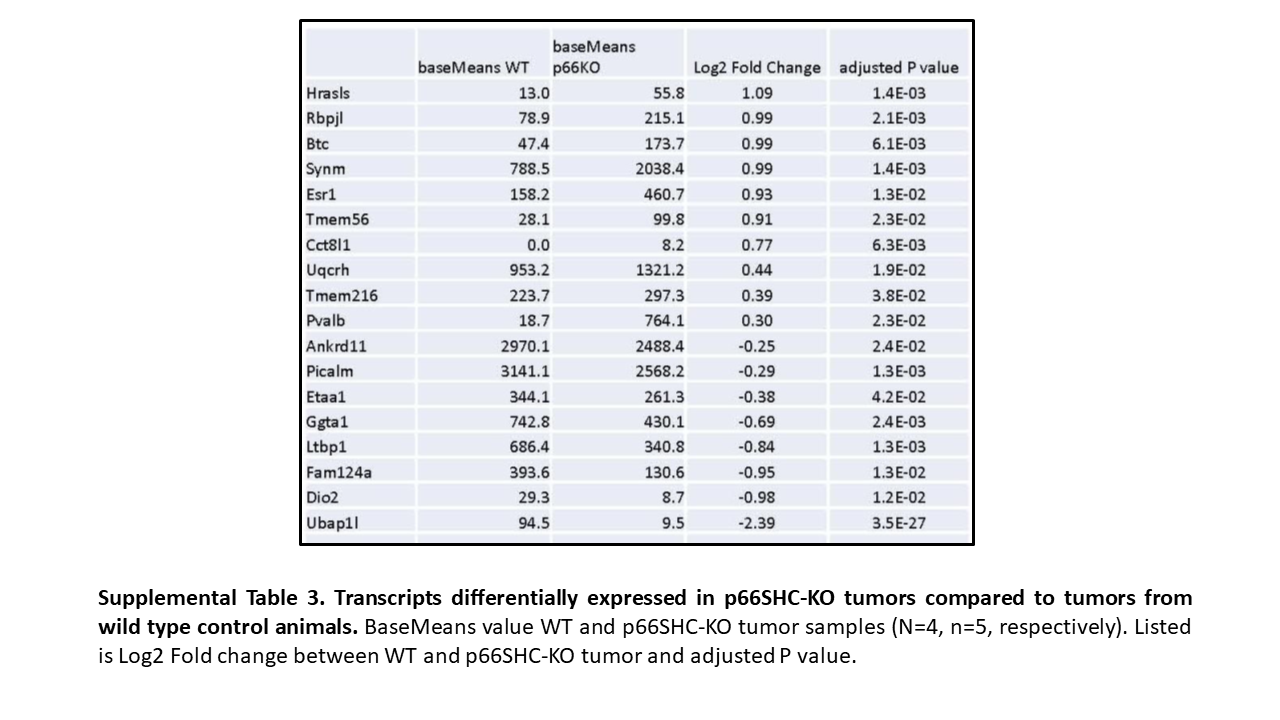

Supplement: Supplementary file 7 — Table S3. Transcripts differentially expressed in p66SHC-KO tumors compared to tumors from wild-type control animals. BaseMeans value WT and p66SHC-KO tumor samples (N = 4, n = 5, respectively). Listed is log2 fold change between WT and p66SHC-KO tumor and adjusted P value. (TIF 345 kb) [file 13058_2019_1155_MOESM7_ESM.tif]
